# Light people: Professor Perry Shum spoke about fiber-enabled industries

**DOI:** 10.1038/s41377-022-00733-8

**Published:** 2022-03-24

**Authors:** Yongkang Dong, Chenzi Guo

**Affiliations:** 1 National Key Laboratory of Science and Technology on Tunable Laser, Harbin Institute of Technology, 150001 Harbin, China; 2 Light Publishing Group, Changchun Institute of Optics, Fine Mechanics and Physics, Chinese Academy of Sciences, 3888 Dong Nan Hu Road, Changchun, 130033 China

**Keywords:** Fibre optics and optical communications, Micro-optics

## Abstract

Fiber technologies have fundamentally reshaped the way we see, the way we sense, the way we communicate, and the way we live. They were so well developed that in some industries such as telecommunication, they were even taken for granted. For that, *Light: Science & Applications* invited Professor Perry Shum, a pioneer in fiber technologies and their industrialization, to speak about what chances fiber technologies can bring to industries.

**Q1:** **The past decades have witnessed the big boom of fiber technologies, and you have pioneered in many of them, such as specialty fibers, fiber sensors and fiber lasers. Could you name the top 3 industries in your mind that fiber-based technologies have brought changes to?**

A1: Top 3 industries in my mind are laser manufacturing, sensing and broadband communication. Ten years down the road, data transmission capacity may increase by tenfold, industries will continue to invest in fiber optics technology because of its reliability and speed of data transfer. With secured and high-speed data transmission being one of our most important requirements today, the future looks very bright for the fiber optics industry.

**Q2: Fiber communication has been a game changer in the field of telecommunication, what are the current major challenges and trend of fiber communication? When can human reach the capacity and transmission distance limit of fiber communication?**

A2: Fiber is the information super highway. However, current fiber optics communication band is limited to C and L Band (i.e., 1525–1625 nm). Since the optical fiber communication was first proposed by Charles Kao in 1966, fiber loss reduced from 1000 dB/km to today’s <0.2 dB/km. In 1987 when David Payne’s research group invented EDFA, we managed to have all basic building blocks ready to make optical fiber communication possible and practical. Another 10 years later, Philip Russell’s research group fabricated the first photonic crystal fiber which makes the design and fabrication of optical fiber three-dimensional and more sophisticated fiber-based devices were being developed. The current major challenges and future trend of fiber communication will be the development of novel fibers to increase the transmission speed as well as data rate. One possible solution is to do away with the fiber core (i.e., coreless). Hollow-core fiber is lossy due to surface mode confinement loss. Anti-resonant fiber is a kind of novel fiber and potentially the fiber loss can be even lower than silica fiber. I predict that the anti-resonant fiber loss may reduce to world record 0.1 dB/km in 3 years. We are still far from reaching the capacity and transmission distance limit of fiber communication. Actually our eyes can see lights from very far away. The most distant known individual star visible to our human eye without any instrument is V762 in the constellation Cassiopeia which is about 16,308 light-years away (1.5 × 10^17^ km).

**Q3: In the past years, many kinds of specialty fibers have been developed, but the common commercial fibers are still dominating the industry, could you share what kind of special fibers might be desired by the industry and predict what kind of specialty fibers can make a real competition to silica fibers?**

A3: Silica fibers will still play an important role in the backbone telecommunication network. Industries are standard driven, conventional single-mode optical fibers are now selling at a price cheaper than noodles in China and the raw material supply silica is sustainable. YOFC is one of the key fiber manufacturers in the world. I visited this company and witnessed how advance this company can draw fibers. Based on the information given, I calculated the speed of making fiber from their 40 m height fiber drawing tower is about 200 km/h. This is almost the speed of high-speed train. Specialty fibers such as biocompatible fibers, microstructured fibers, multifunctional fibers, THz fibers, mid-IR fibers, nano-fibers, bandgap fibers and imaging fibers will be needed for specific applications.

**Figure:**
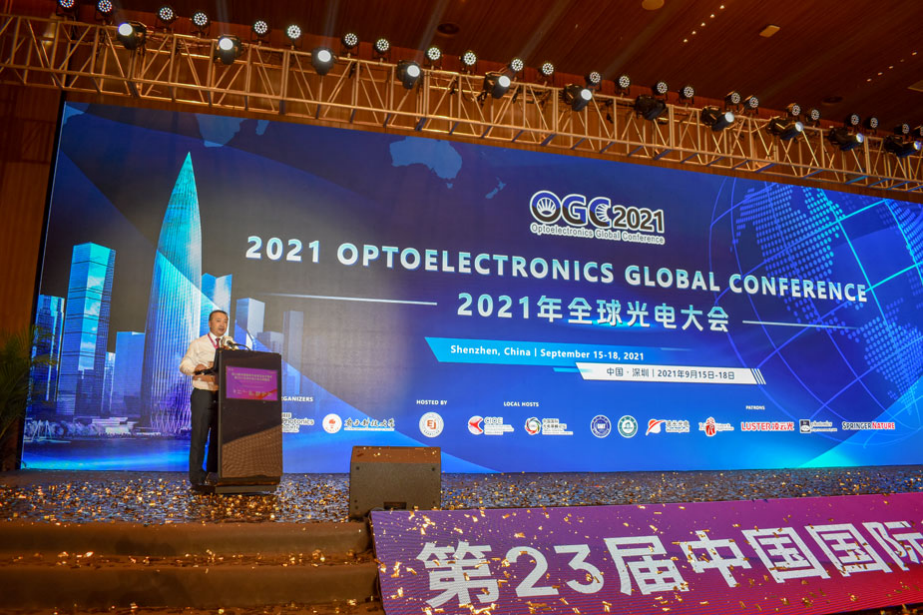
Prof. Shum chaired the OGC 2021

**Q4: In the past 10 years, distributed acoustic sensor (DAS) has experienced a prosperous development. Can you comment on its significance and application in various fields?**

A4: The advantage of distributed acoustic sensor (DAS) is its fast distributed measurements over long distances. We can achieve large-scale distributed acoustic detection based on the phase-sensitive time-domain reflectometry (ϕ-OTDR). DAS can find applications in various scenarios such as geological sensing, oil and gas security, intrusion detection and railway infrastructure monitoring. In recent years, many advanced techniques are being developed to enhance the performance of ϕ-OTDR sensors. Key considerations include the reduction of measurement noise, the increase in the dynamic measurement capacity, the sampling for multiple perturbations and the enhancement of the measurement spatial resolution. There is a bright future for the implementation of DAS in safety, security and integrity monitoring systems.

**Q5: Fiber lasers have revolutionized manufacturing industry, and become the mainstream laser manufacturing technique. Looking into the next decade, what are the trend of fiber lasers, and do you think any kind of lasers can compete with or even surpass fiber lasers, in terms of CW power and generating high-performance ultrashort pulses, and upgrade the industry of manufacturing?**

A5: Fiber lasers have many special properties such as high power generation, good reliability, compact size, low noise and good beam quality. Fiber lasers can be used for many applications such as welding and cutting, marking, printing, micromachining, drilling, soldering and thermal annealing. Ten years down the road, there will be continuous demand for CW fiber laser with higher power. For the removal of material without incurring heat (i.e., avoid melting or cracking), we will need to use femtosecond or picosecond laser pulses to carry out athermal ablation. The trend for next decade would be the development of pulsed fiber laser with high average power and short pulse duration. Ultrafast laser applications include life sciences, energy and advanced materials fields. One of the competing technologies could be disk laser which demonstrated the advantage of both high pulse energy and high average power. Disk lasers are considered solid-state lasers consisting of a thin slice of ytterbium-doped yttrium-aluminum garnet crystal. In order to develop low-cost short pulse fiber laser, we have to investigate efficient power combiners as well as high power optical amplifiers suitable for different wavelengths.

**Q6: Fiber-enabled endoscopy has been extensively studied. Compared to other in situ sensing technologies, what strengths and weaknesses does fiber-enabled endoscopy have?**

A6: Endoscopy has two broad application areas: industrial and medical. In medical applications, fiber-enabled endoscope is flexible with regard to rigid endoscopy, allowing access to non-straight lumen and cavities. With regard to endoscopy without fiber, such as capsule endoscopy, fiber-enabled endoscopy has the advantage of compact and higher excitation light power and allows distal end photodetector. Note that distal end photodetector is much more powerful than the proximal end detector used in capsule endoscopy. With fiber, therapy such as laser ablation with high power laser is possible. The weaknesses of fiber in endoscopy are all minor and application dependent. This optics-based technique would not be suitable when wireless applications are needed.

**Q7: You have been well known as a great educator. Some of your students have not only established themselves as leading scientists, but also built up listed companies. Could you shed insight into how to guide different students to what suits them best?**

A7: I consistently supervised about six PhD students when I was in Singapore, that’s the maximum number I can manage. Though I supervised students from all around the world, the amount of Chinese students dominated my group. Many of them have later returned to China and brought huge input into the industries. There was one special student who did PhD with me with a clear intention to start up a hi-tech company. His objective was clear and his company is already a listed company. There was later another bright student I knew he would be very successful if he startup a company. His objective was not quite clear at the beginning so he decided to work in a research lab for a couple of years. He then decided to start up a company and I think his company will be the next listed company sooner or later. Apart from my own students, I also assisted many young researchers at their early stages of starting up companies. All of them happened to be doing so well and I have to seek for their help from time to time to solve my own technical problems at lab. There is no general formula but I am happy to share my recipes. For those talented and determined students, we should give them minimal supervision and try to give high-level advices. Most of our time should be given to students who have not found their ways to researches or lives. Good to maintain a close contact with our students and try to create opportunities for them to move up the corporate ladder when needed.

**Figure:**
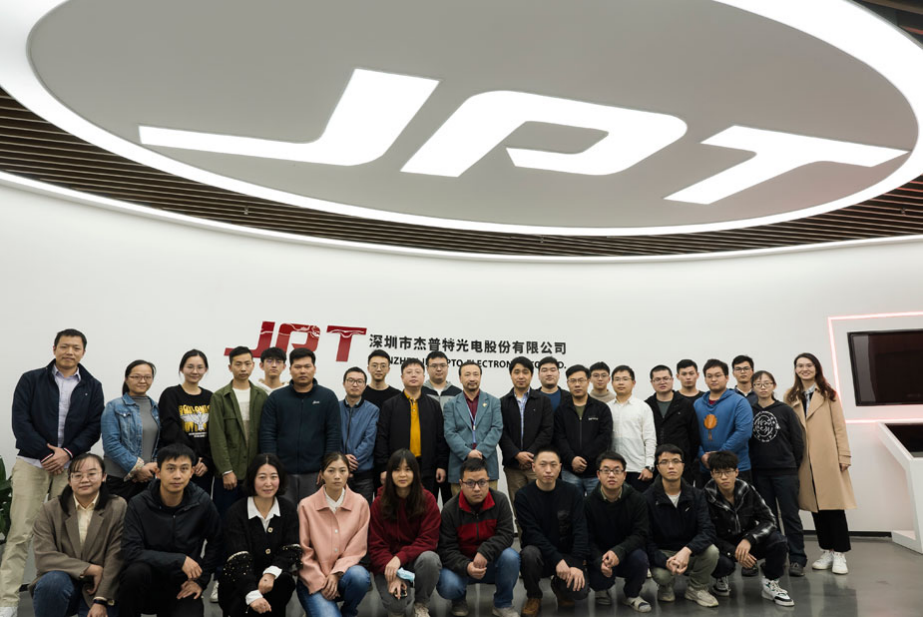
Prof. Shum’s group photo with JPT R&D team

**Figure:**
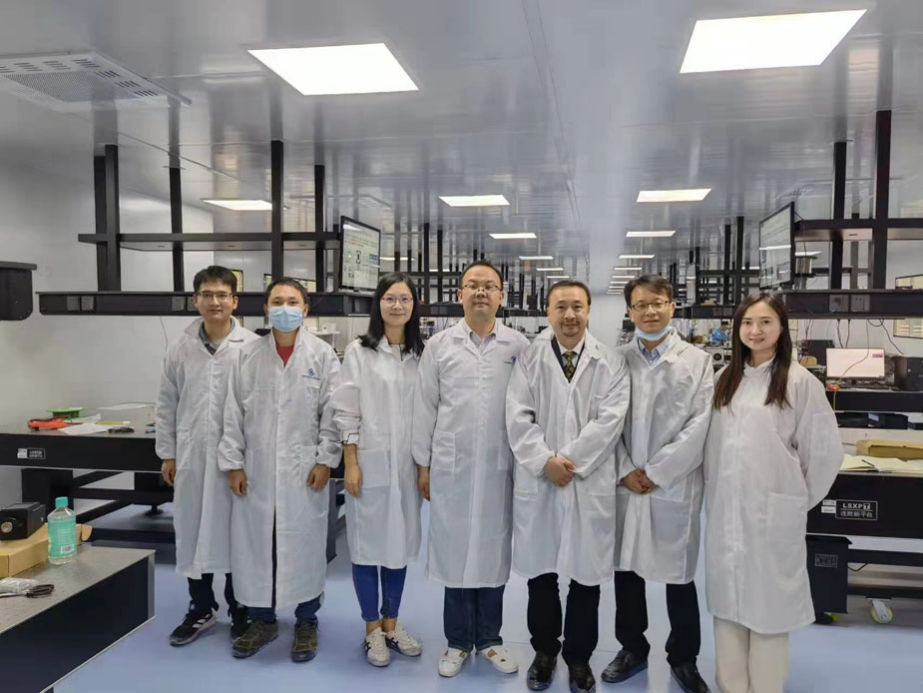
Prof. Shum visiting Professor Songnian Fu’s new lab in Guangzhou

**Q8: Once we talked about one of your students who spent so many years struggling in science and almost lost confidence in everything, but you managed to get him through. Could you share some words with those students who may doubt themselves at the very beginning of their researches?**

A8: Basically all my PhD students graduated in about 3–4 years and there was a single student who broke the record and graduated in 2.5 years by doing a part-time PhD. The student I mentioned to you was an undergraduate student. He came to me for final year project (FYP). When I accepted him, I knew that he had troubles with many undergraduate courses which he should have completed. His unconfidence was transplanted from his study to his daily life, so severe that he lost control of self-management and gained a lot of weight and was unable to face his parents in person over the past 2 years. At that time, he was obliged to finish both the remaining courses and FYP within a year; otherwise, he would end up with nothing and no bachelor degree. With his situation, my conscience left me no choice but to work out a solution together with him.

First, I encouraged him to take my class (Optoelectronics Intellisense) so that I can have better idea about his study habit and question answering ability. Meanwhile, I took photos of him and sent to his parents (secret mission), so that his parents can help encourage him. Later, I changed his FYP to something more relevant to his interest. Therefore, the final topic of his FYP was no longer photonics project but developing an Escape Game mobile App. Through the process, I helped him to rebuild the confidence in his own intelligence, and work out time management skills. He did extremely well in his FYP and one of his examiners told me that he was one of the best students in the final project presentation. He finally graduated smoothly and my mission was basically accomplished. However, he was not interested in finding a job after graduation. I then convinced him to gain some job experience despite all excuses. I was very lucky in the past 20 years that many good students came and study with me. By supervising this student I learned some students suffer because they are not aware of the difference between interest and curiosity. Most of the time there is a steep learning curve and the journey could be arduous before we can claim that we have real interest in something. Some students may choose to give up and the easiest way is to quit. They can always find many excuses to justify their failure but how many of them willing to say “sorry” and accept that there is room for self-improvement. There is a very nice Chinese word “感恩” which means gratefulness or thankful. We should feel grateful for all the difficulties or bad lucks we have gone through in life. We should feel thankful for all the mentors we met and treasured the love and kindness from parents. Try not to be so stubborn too early. My definition of stubbornness is someone having problem with the receiver. Therefore, this person needs much stronger amplified signals from various sources in order to receive information. My advice for students is to open your arms for changes. It’s difficult for us to make changes if we do not listen and digest useful knowledge. The man with wisdom knows the importance of using their receivers (i.e., listening) while some people using their transmitters (i.e., talking) all the time. Time is an invaluable parameter. Some people used too much time and health to earn money when they were young. When they are getting old, they have to return the money in exchange of their health but not the time. When we are young, try to learn from senior professors with a humble heart. One day we become senior scientists, we should try to help junior researchers as much as we can if a mutual trust can be established. When we are getting old, all these junior researchers may achieve much more than us and we should feel proud on what we contributed to the society. They would remember your birthday and call you mentor or yyds.

**Figure:**
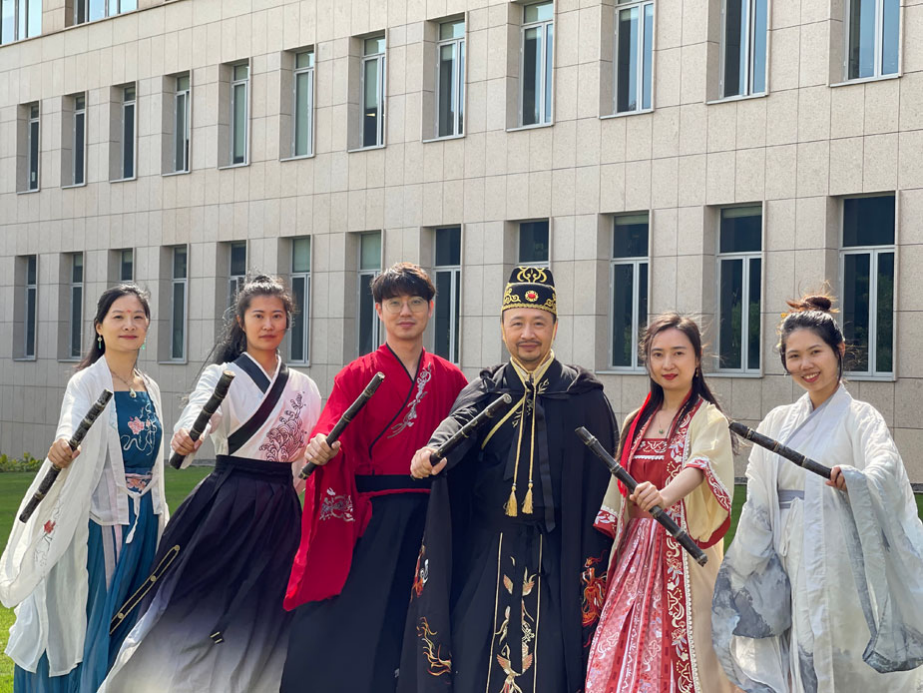
Prof. Shum and his group members cosplayed in China’s traditional costumes

**Q9: You have contributed a lot to** ***Light: Science & Applications*, how do you think the journal can interplay more with fiber-related researches and industries?**

A9:LSA can enhance its influence in fiber-based technologies by organising special issues or review papers on fiber laser, fiber sensor or disruptive fiber-based technologies. I suggest LSA to host special sessions or workshops in some major conferences locally or internationly. In my own capacity I can help to organise events during CIOE or ACP this year in Shenzhen. This year is the fifth International Day of Light (IDL), we can organise special LSA event on 16 May or during the month of May. John Dudley is the key member to make International Year of Light as well as IDL possible. He used to keep a journal special issue on Optical fiber-based devices and applications in his briefcase. This special issue was edited by me, Jonathan Knight, Jesper Laegsgaard and Dora Hu in 2010. John told me he enjoy reading the papers in that special issue because most of the papers are relevant to his interest. As you know, we're planning with LSA to setup a regional office in Shenzhen, this new office would help to reach out to photonics industries and researchers in the region. I hope we all can work closely with LSA and reach another new height together.

**Short Bio**

Dr Prof. Shum Ping is Chair Professor of Southern University of Science and Technology (SUSTech), OSA Fellow, SPIE Fellow, and chairman of IEEE Photonics Society Guangdong Chapter. In previous 3 years, he served as the Vice President of IEEE Photonics Society. His research interests focus on optical fiber technologies, optical sensing, laser technologies, silicon photonics and biomedical photonics including Optical Coherence Tomography, Raman spectrum imaging, Surface Enhanced Raman Scattering, multi-omics based deep learning for the applications of cancer early/precise diagnosis, prognosis and optimal therapy decision. He has published more than 1000 peer-reviewed papers with an H-index of 62, and served as the chair of many major conferences including CLEO-PR, OECC, ACP, PGC, OGC, ICOCN, ICAIT, etc. Prof. Shum has created the special project *Enabled Learning: Escape Room Design*, which has been reported by Channel News Asia, Channel U, Channel 8, Channel 5, Zhaobao, in four different languages. He has also cultivated many distinguished students, some of them are founders of high-tech companies such as Raintree Photonics and JPT Optoelectronics.

**Figure:**
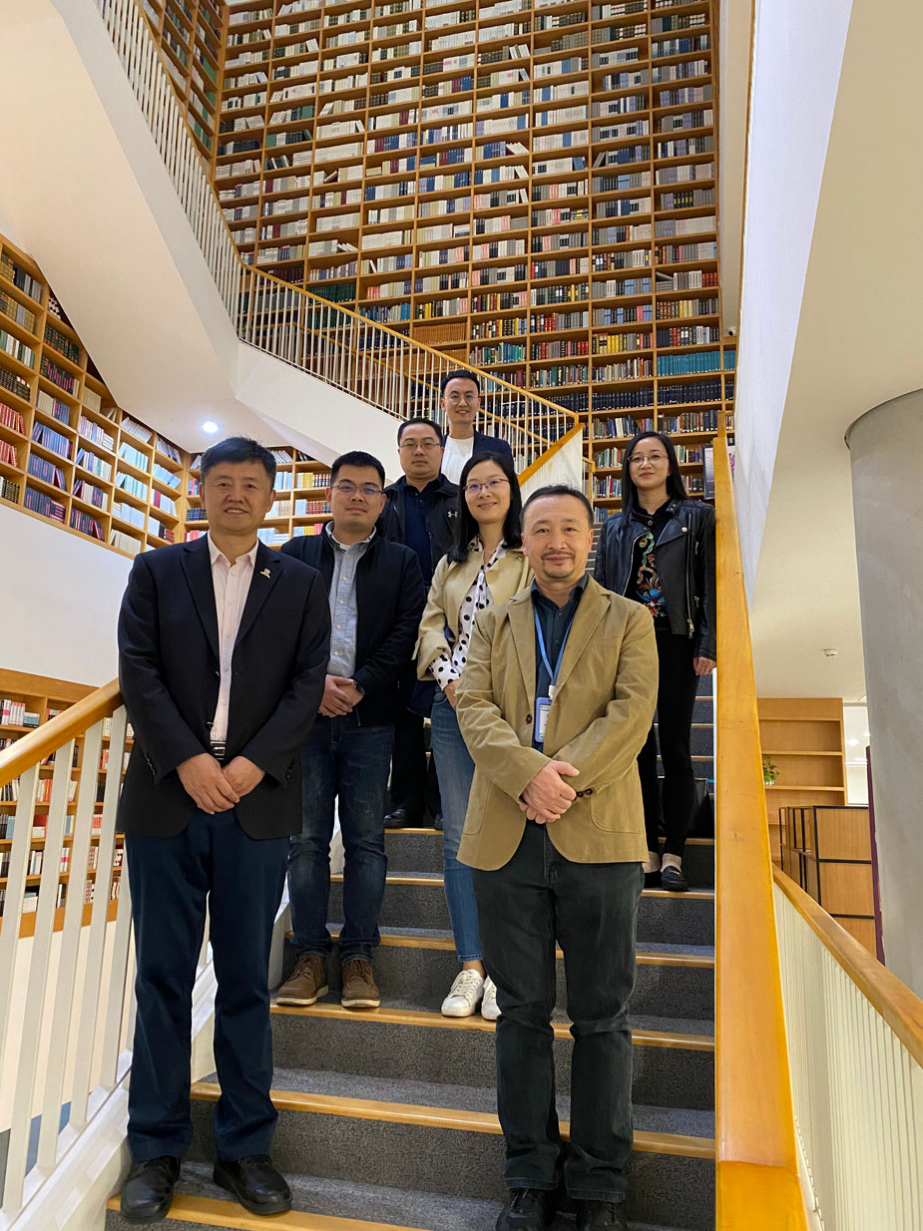
IEEE Photonics Society Guangdong Chapter 2022 committee

